# Functional and Structural Uterine Changes in PCOS

**DOI:** 10.3390/ijms26167921

**Published:** 2025-08-16

**Authors:** Lucja Zaborowska, Joanna Maria Blok, Emilia Piotrkowicz, Steven R. Lindheim, Artur Ludwin

**Affiliations:** 1 Doctoral School of Medical and Health Sciences, Jagiellonian University Medical College, 31-008 Cracow, Poland; 2 1st Department of Obstetrics and Gynecology, Medical University of Warsaw, 02-015 Warsaw, Poland; 3 Department of Obstetrics and Gynecology, Boonshoft School of Medicine, Wright State University, Dayton, OH 45409, USA; 4 Ludwin & Ludwin Gynecology, Private Medical Center, 31-510 Cracow, Poland; 5 Centermed Hospital, 31-530 Cracow, Poland

**Keywords:** uterine morphology, uterine blood flow, polycystic ovary syndrome, uterus, animal studies, pregnancy, uterine function

## Abstract

(1) Polycystic ovary syndrome (PCOS) is one of the most common endocrinological disorders worldwide; its complex etiopathology remains poorly understood. PCOS is associated with a broad spectrum of abnormalities, including irregular menses, androgen excess, and increased risk of metabolic, endocrinological, and cardiovascular disorders. This narrative review focuses on structural and functional changes in the uterus associated with polycystic ovary syndrome and hyperandrogenism. (2) The review was performed by searching PubMed, Medline, Embase, Google Scholar, and Cochrane Library electronic databases on records published between 1964 and 2025. The authors included studies on (i) the uterus in clinical settings of PCOS patients, (ii) the uterus in PCOS models, and (iii) the pregnant uterus in patients with PCOS. Multiple animal and human studies describe a potential impact of PCOS on uterine blood flow, morphology, and thickness of the uterine muscle, indicating a possible functional impairment in pregnant and non-pregnant women. The scope of available knowledge regarding functional and structural uterine changes in PCOS is scarce; new studies are warranted. Future research should focus on hyperandrogenism associated with PCOS and explore the link between the morphology and function of the uterus.

## 1. Introduction

Polycystic ovary syndrome (PCOS) is one of the most common endocrinological disorders affecting women of reproductive age [1]. Its etiology is poorly understood and combines genetic, epigenetic, intrauterine, and environmental factors [1,2,3]. PCOS and its impact on fertility have been widely studied and documented, especially regarding morphological changes in the ovaries and hormonal imbalance, leading to specific disease symptoms [4,5,6]. Until recently, the uterine aspects of PCOS have been widely disregarded [7,8,9,10,11]. The possible variations in morphology, function, and blood circulation may play an essential role in the spectrum of subfertility described in PCOS women. The morphological changes associated with prolonged hormonal imbalance accompanying PCOS are currently not widely recognized. The clinical significance of these outliers is mostly unknown, although some uterine malformations have already been linked to recurrent miscarriages, preterm labor, infertility, and increased assisted reproductive technology use [12,13].

Here, the authors present a narrative review of the data on the function and morphology of the PCOS uterus. This study aimed to (i) assess the current state of knowledge regarding the impact of PCOS on the uterus in clinical trials, animal models, and pregnancy, and (ii) determine data gaps and new outliers for further research. The article discusses clinical and preclinical trials focused on different features of PCOS and their possible link to uterine function and morphology.

## 2. Materials and Methods

The review was performed by searching PubMed, Medline, Embase, Google Scholar, and Cochrane Library electronic databases on records published between 1964 and 2025, using keywords “PCOS”, “hyperandrogenism”, “uterus”, “measurements”, “morphology”, and “function”, as well as their synonyms and variations. The search was performed simultaneously by two authors (LZ, JB). All possible discrepancies were resolved by a third author (AL). The search strings were modified according to each database’s model. There were no language restrictions, although the authors utilized English keywords. All articles were screened for the title and abstract. The authors additionally performed the reference search of included articles. Included research tackled the subject of uterine morphology or uterine function directly linked to its morphology assessed via ultrasound: shape, dimensions, volume, fibroids, endometrial thickness, and blood flow. The primary outcomes were divided into three groups of interest: (i) the uterus in clinical settings of PCOS patients, (ii) the uterus in PCOS models, and (iii) the pregnant uterus in patients with PCOS. The authors included observational, clinical, and model studies, as well as reviews, to ensure the complexity and novelty of the described data.

## 3. General Background

### 3.1. Polycystic Ovary Syndrome

The disease presents with varying symptoms including hyperandrogenism and impaired ovulation, caused by a complex fusion of ovarian, metabolic, and pituitary dysfunction. A model hormonal imbalance manifests with a rise in LH secretion and lower FSH concentration. Various metabolic changes are a common associated feature [14,15,16,17].

Multiple studies have suggested a genetic background of the disease [3,18]. The suspected inheritance pattern was further validated using twin studies, advocating over 70% influence of familiar factors in the pathogenesis of the disorder [19]. A nationwide register-based cohort and a clinical case–control study provided evidence of a fivefold-increased risk of PCOS for daughters born to women with PCOS, as compared to mothers without the disease [20].

#### 3.1.1. Diagnosis

Diagnosis in adults is typically made using the Rotterdam 2003 consensus criteria [21], covered within three crucial aspects of the disease: (I) oligo- or anovulation, (II) clinical and/or biochemical signs of hyperandrogenism, and (III) polycystic ovaries. Only two out of three criteria need to be fulfilled simultaneously.

I. Ovulation disruption can be confirmed by either irregular periods or regular, anovulatory cycles confirmed via progesterone serum concentrations [22,23].
II. Hyperandrogenism can be assessed in both clinical and biochemical manner—the clinical features include, e.g., hirsutism evaluated with the modified Ferriman–Gallwey score, alopecia, and severe acne. Biochemical indicators include the free androgen index and calculated free or bioavailable testosterone.
III. Polycystic ovaries are described as more than 20 coexisting pre-antral follicles, measuring 2–9 mm in diameter, present in either ovary and/or an increased ovarian volume > 10 cm^3^. The number of follicles is an update of 12 reported in the original consensus from 2003 [23].

A complete diagnosis requires the exclusion of other etiologies leading to experienced signs and symptoms, such as congenital adrenal hyperplasia, hyperprolactinemia, androgen-secreting tumors, or Cushing’s syndrome. Assessment of hyperandrogenism should not be performed when one is using hormonal contraceptives; a 3-month withdrawal period is the recommended minimum [22,23]. As previously mentioned, the concentration of LH and FSH might indicate PCOS, with the elevated LH/FSH ratio present in up to 95% of non-ovulating patients [24]. However, measurements of both these pituitary hormones should not be considered a crucial part of the investigation. Diagnosis in adolescents should be made with caution.

#### 3.1.2. Clinical Features

PCOS should be considered in all females with clinical features of hyperandrogenism, including hirsutism, alopecia, irregular menstruations, acanthosis nigricans, or severe acne. Obesity is a common finding, although it does not constitute a part of the diagnosis. Despite this, insulin resistance is detectable in 2/3 of women affected and is irrespective to their reported BMI [15,25]. Women with PCOS usually present with high concentrations of serum Anti-Müllerian Hormone (AMH) [26,27]. A recent guideline underlined the significance of its levels, considering them as a substitute for the ultrasound ovarian assessment [16]. PCOS risk groups include patients with early adrenarche, obesity, insulin resistance, diabetes mellitus (type I, II, gestational), family history of PCOS, and use of antiseizure medications [3,28,29,30].

There are four distinguishable phenotypes of PCOS, emerging from the combinations of 3 Rotterdam consensus criteria; type A encompasses all aspects of the disease, while type D is the only one that does not incorporate hyperandrogenism.

#### 3.1.3. Pathogenesis

The intergenerational transmission of PCOS seems to validate the theory of fetal origin of the disease and suspected prenatal exposure to androgens and AMH [20,31,32]. High AMH levels contribute to lower testosterone metabolism within the placenta, resulting in the masculinization of exposed individuals [32]. A linked theory that could further explain the early onset of symptoms revolves around the inherited, genetic background of the disease [3,18]. The role of prenatal androgen exposure in the pathogenesis of PCOS has been a subject of considerable interest and investigation. It is hypothesized that excessive exposure to androgens during intrauterine life may disrupt normal fetal development, leading to the manifestation of PCOS traits later in life. One of the studies assessing the impact of maternal hyperandrogenism during pregnancy reported significantly higher levels of testosterone in umbilical vein blood collected after delivery of the placenta in PCOS women compared to the healthy controls [33]. Furthermore, a small prospective case–control study showed that fetal exposure to excess androgens of PCOS mothers may result in higher sebum production in newborns [34].

Studies performed on pregnant animals show that testosterone hyper-exposure during pregnancy might cause polycystic ovaries, high sebum, LH concentrations and insulin resistance in the offspring [35,36,37,38]. Furthermore, a 2006 case–control [39] and a 2010 cohort study [40] showed that raised maternal androgens during pregnancy may impact the ovarian function of female offspring by increasing AMH levels in adolescence. AMH is a hormone produced exclusively in gonads, responsible for the regulation of early follicle growth [41]. AMH levels are elevated in reproductive age women with [42,43]. This suggests that hyper-exposure to androgens in utero might cause increased serum AMH concentration in daughters of women with PCOS, followed by altered follicular development However, significantly higher levels of androgens do not appear to result in fetal virilization [2]. This is due to multiple fetal protective mechanisms, such as converting maternal and fetal androgens to estrogens by the increased placental aromatase activity, increase in maternal circulating sex hormone-binding proteins (SHBG), or decrease in testosterone conversion [44,45,46]. The pregnancy itself is known to mitigate symptoms of hyperandrogenic disorders [47].

A set of pathomechanisms that accompany PCOS includes insulin resistance [48]. Greater circulating insulin levels promote increased androgen concentration by stimulating their production in ovarian cells and inhibiting the SHBG [18,49]. Some researchers emphasize the role of adipose fat tissue in converting and synthesizing androgens [50], which may further intensify the syndrome’s features. Typical features of PCOS, increased secretion of LH and its subsequent imbalance to FSH promote the secretion of androgens in ovarian follicles. Due to the relative deficiency of FSH, the selection of a dominant follicle is impaired, leading to even further production of androgens and the creation of multiple enlarged follicles visible during ultrasound inspection. The lack of the dominant follicle and its rupture results in lower progesterone levels, typically elevated during the second part of the menstrual cycle. An insufficient amount of progesterone cannot inhibit gonadotropin-releasing hormone, resulting in further production of LH and FSH and repetition of the entire process [51].

#### 3.1.4. Comorbidity

Patients with PCOS are at greater risk of developing insulin resistance, metabolic syndrome, and diabetes mellitus type II [48,52,53,54]. The syndrome is also connected to a higher incidence of cardiovascular complications [55]. The relative scarcity of progesterone caused by impaired ovulation results in excessive circulating estrogens that may manifest, e.g., with the development of endometrial adenomatous hyperplasia and cancer [56,57].

Pregnancy complications, such as premature birth, miscarriage, preeclampsia, and gestational diabetes mellitus, are significantly more common in women with PCOS [6,58,59]. The spontaneous abortion rate among these patients is up to 40% higher than in the general population [59].

### 3.2. Uterus

The uterus is a singular female reproductive organ located between the rectum and the bladder, encompassed within the bones of the pelvis. The uterus arises from Müllerian ducts, guided by the Wolffian (mesonephric) ducts. Since the mesonephric duct plays a role in the formation of the urinary system, the coexistence of congenital uterine and renal anomalies is often described [60,61,62]. The Anti-Müllerian Hormone (AMH) drives the process of sex differentiation by facilitating the regression of Müllerian ducts in males. In female embryos, AMH is not produced until the late stages of the pregnancy, allowing for uninterrupted evolution of the female reproductive tract [63].

The uterus begins developing around the 8–10th week of the pregnancy, along with the fusion of two Müllerian ducts [64,65]. At the very beginning, the epithelial septum separates two organ cavities. The separation regresses around the 20th week of gestation [66,67]. At 22 weeks, we are able to distinguish the uterine tube, corpus, cervix, and vagina [65]. Various factors may disturb Müllerian ducts’ development and lead to different fusion defects. These variables include diethylstilbestrol (DES), a synthetic estrogen associated with various uterine anomalies after in utero exposure [68]. Some authors have presented the androgen-dependent theory of uterine anomalies and linked it to the Hox gene that may affect the development of Müllerian ducts [69,70,71].

## 4. PCOS in Clinical Trials

### 4.1. General Uterine Morphology Asessment

The etiology of uterine anomalies is not fully understood but may arise from various genetic, environmental, and developmental factors [72]. In multiple available studies, PCOS was associated with higher incidences of congenital uterine malformations [69,70,73,74]. The scope of possible pathophysiology includes prolonged androgen exposure in utero and elevated levels of AMH—both of which can possibly alter the development of Müllerian ducts [63,75,76]. A recent systematic review has confirmed a higher incidence of uterine anomalies in PCOS setting [77]. The generalizability of these results is limited by the very low quality of available evidence. Septate, I-shaped, dysmorphic and didelphys uteri were listed among anomalies that may be connected to the hormonal disturbances in PCOS [77]. Analogously, fundal indentation depth and angle were found to be significantly altered in PCOS patients. Only one study explored the possible link between hyperandrogenism and observed uterine changes [77].

Recent studies have used 3D-US as a reliable tool for diagnosing uterine malformations in PCOS settings [10,78,79]. However, most research continues to utilize the 2D technique, with magnetic resonance imagining, hysterosalpingography, hysteroscopy, or laparoscopy as a confirmation method. Two-dimensional ultrasound is characterized by unknown and limited accuracy in assessing minor and major uterine malformations, respectively [12,80]. Its reliability in the proper classification of anomalies of the reproductive tract is doubtful. Moreover, both hysteroscopy and laparoscopy exhibited inferior reliability in differentiation between basic uterine morphologies, i.e., normal, arcuate, and septate uterus [81,82,83,84]. It is worth noting that these minimally invasive methods do pose significant discomfort for affected patients.

### 4.2. Fibroids

Fibroids (leiomyomas) are benign neoplasms of the uterus. The prevalence of leiomyomas increases with age, up to menopause [85]. They seem to be more common in women with greater BMI [86,87]. Fibroids are hormone-dependent—their tissue is characterized by an overexpression of estrogen and progesterone receptors [88,89]. On the other hand, these tumors tend to have an upregulation of aromatase, an enzyme converting androgens to estrogens [90,91].

A secondary analysis of data from three randomized clinical trials revealed that infertile oligo-ovulatory women with PCOS had a reduced prevalence of non-cavity-distorting fibroids compared with regularly ovulating women with unexplained infertility [92]. The results may differ for fertile reproductive-age women, so caution should be exercised in drawing firm conclusions about the general population [92]. This finding is consistent with previous studies that suggested PCOS may have a protective effect on development of leiomyomas [93,94]. However, this differs from a report by Wise et al. that found a 65% increased risk of fibroids among Black women with PCOS [95]. The difference between the results might be attributed to multiple factors: method of screening, ethnicity of the subjects, or fibroid types included in the study.

### 4.3. Endometrial Thickness

It is believed that the endometrial thickness in women with PCOS is generally greater than that in a healthy population, which can be a sign of irregular menstruation and anovulation. These changes are believed to occur due to a relative excess of estrogens in PCOS, which can eventually lead to abnormal hyperplasia of the endometrial lining [56,57]. Patients affected by the disease have up to 8.42 times greater risk of endometrial cancer compared to a healthy population [96]. Interestingly, a prospective study of 1198 patients found no statistical difference between endometrial thickness in PCOS women and healthy controls [97]. The uterine lining was measured via the transvaginal ultrasound (TVUS) on the 3–7th day of the menstrual cycle [97].

A few older, prospective case–control studies replicated these results, showing no statistical difference in uterine lining in follicular phase [98] and significantly decreased (thickness in both peri-ovulatory and luteal phase in the PCOS group [99]. Another prospective case–control study from 2012 detected a thinner endometrium in women with PCOS and oligo-amenorrhea than in healthy controls [100]. The examination was performed between the 11–17th day of the cycle and was assessed both via magnetic resonance imagining (MRI) and TVUS. Additionally, a small study from 2006 compared women with insulin resistance with and without PCOS and reported significantly thicker endometrium in the PCOS group [101]. This finding was based on three consecutive TVUS scans performed between 6 and 10th days of the menstrual cycle.

Henceforth, due to the heterogeneity of used methods and acquired reports, more research is needed to draw the definitive clinical implications of the results.

### 4.4. Myometrial Thickness

Previous studies conducted on a limited number of patients have not shown significant differences in myometrial thickness between PCOS and healthy subjects [100]. However, in a recent cross-sectional study by Fujii et al., myometrial thickness was shown to be significantly reduced in PCOS women compared to healthy controls [70]. Additionally, some authors demonstrated growth of uterine muscle after treatment with an oral estrogen/progestogen combination [102].

### 4.5. Uterine Arteries

Uterine artery velocimetry in PCOS, performed both in 2D and 3D Doppler, has been proposed as a one of possible non-invasive indicators of endometrial receptivity. These measurements are thought to act as a predictive value for implantation success—even if some studies do not report any difference between tested groups [103,104].

A prospective study of eighty-eight PCOS-affected patients and 15 controls confirmed that patients with PCOS have higher resistance in uterine arteries than those without PCOS [105]. A few years later, another study reported a significantly lower endometrial and subendometrial blood flow in PCOS patients compared to controls. The authors also noted a significant improvement accompanying a metformin treatment [99]. These findings were only partially confirmed by newer studies performed with the use of 3D ultrasound. According to the study by Lam et al., only hyperandrogenic women with anovulatory PCOS had significantly lower endometrial and subendometrial blood flow than normal controls [98]. A similar study published in the same year found that uterine artery pulsatility index (PI) and resistance index (RI) were significantly increased [106]—these results were consistent with baseline values reported for 18 patients in a 2006 intervention study [107]. Moreover, it was established that PCOS does not predetermine a single uterine blood flow pattern—a wide range of PI values can be observed [106]. Higher BMI and DHEAS seem to influence the uterine artery’s pulsatility index in PCOS patients [105,108]. On the other hand, increased body weight negatively affects uterine perfusion and worsens endocrinological and clinical patterns. Another factor that may alter uterine perfusion can be adrenal derived DHEAS; its excess has been attributed to adrenal androgen hyperresponsiveness or intrinsic adrenal dysfunction in PCOS patients.

Available studies on uterine blood flow are presented in Table 1.

## 5. PCOS in Animal Models

### 5.1. Uterine Morphology

A recent article presented an experimental mode study that generated a hyperandrogenized environment of PCOS in 21 female rats via subcutaneous injections of DHEA for 20 consecutive days [109]. After the excision, the weight of the body of the uterus was measured before and after drying the tissues and then analyzed. The examination showed significantly increased weight and diameter of the uteri in rats of the research group. Increased thickness in the tissue compartments was proved, which was associated with a reduction in cell density and increased water content in both [109]. Alternatively, Ferreira et al. studied the influence of the prenatal hyperandrogenic environment on the uteri of adult rats. Female rats were administered subcutaneous injections of testosterone during pregnancy for 4 consecutive days; the obtained litter was compared to a non-prenatally androgenized litter serving as the control group. The efficacy of reproducing a PCOS environment in a litter during puberty and adulthood was evidenced by the high levels of serum testosterone. The total uterine thickness was increased in the prenatally hyperandrogenized group when compared to the control group. No difference between groups were noted regarding the thickness of the glandular epithelium [110]. The increased number of glands and the occurrence of gland conglomerates were evidenced in the endometrium of prenatally hyperandrogenized groups. Thus, the rodent model of prenatal hyperandrogenization showed abnormal morphology of the uterus including endometrial hyperplasia and disturbance in the cell cycle of the uterine [110].

Additionally, in a DHEA-induced PCOS mouse model, transferring blastocysts from the control group mice into the uterus of pseudopregnant PCOS individuals resulted in a reduced rate of embryo implantation due to dysregulation of the implantation-related genes [111]. More recently, a 2023 study relied on a mouse model with PCOS generated by 20-day-long DHEA subcutaneous administration [112]. The research used impaired the estrous cycle to confirm the successful reproduction of a PCOS environment. The results showed a statistically significant difference in mean endometrial thickness—the DHEA group displayed a thicker endometrium with signs of hyperplasia [112].

### 5.2. Gravid Uterus in PCOS

In a 2019 animal model study, simultaneous exposure to 5α-dihydrotestosterone (DHT) and insulin (INS) during gestation caused uterine and placental defects due to disrupted reactive oxygen species (ROS) production and inactivation of anti-oxidative proteins [113]. Few of those anti-oxidative proteins, namely, nuclear factor erythroid 2-related factor 2 (Nrf-2) and superoxide dismutase 1, inhibit ferroptosis—an iron-dependent form of regulated necrosis induced by oxidative stress [114,115]. One of the first studies exploring the relationship between increased DHT and INS levels and activation of ferroptosis in the gravid uterus was performed in 2000 [116]; the authors showed that ferroptosis in the gravid uterus occurs due to decreased GPX4 and glutathione abundance, altered expression of ferroptosis-associated genes (Acsl4, Tfrc, Slc7a11 and Gclc), increased MDA and iron deposition, upregulation of the ERK/p38/JNK pathways, and mitochondrial Dpp4 expression [116]. Furthermore, in PCOS women, several mitochondria abnormalities have been shown to be responsible for disturbance in the regulation of ferroptosis [117]. Five ferroptosis-related genes have been identified: NOX1, ACVR1B, PHF21A, FTL, and GALNT14 [118].

Ferroptosis have become a spotlight for developing new methods of long-term PCOS treatment, with potential targets including iron metabolism and circRNA [119]. A combination of rat and in vitro PCOS models showed that cryptotanshinone has an inhibitory effect on cellular ferroptosis and could be proven effective in the treatment of PCOS [120]. Furthermore, inducing ferroptosis via upregulating TFR1 expression may represent a promising strategy for managing ovulation in PCOS women [121]. Future potential treatment targets for PCOS patients include circular RNA and transferrin receptor signaling that may further affect ferroptosis and folliculogenesis [121,122].

## 6. The Impact of Hyperandrogenism on a Gravid Uterus

PCOS is typically characterized by impaired ovulation; affected women often present with accompanying obesity, insulin resistance, and metabolic abnormalities, which might contribute to the increased risk of obstetric and neonatal complications [123,124]. Moreover, many PCOS patients with maintained spontaneous ovulation are infertile, which suggests that other maternal and fetal factors might severely impact their reproductive outcomes [125].

After the 28th week of pregnancy, a physiological increase in the level of androgens occurs [126]. The significance of this finding remains unknown [127,128], but multiple studies suggest that androgens induce a relaxation of contractile activity in the human myometrium [129,130]. Although the physiological increase in androgen levels seems to have pregnancy-maintaining properties, androgen excess in PCOS patients is considered an important risk factor for pregnancy complications [128]. Mechanisms protecting the fetus from androgen excess include androgen metabolism by the aromatase complex within the placenta [44] and increased levels of plasma SHBG.

### 6.1. Endometrial Component of Subfertility in PCOS Patients

The endometrium is a well-known source of angiogenic factors, initiating the process of neovascularization in response to hypoxia [131]. Endometrial angiogenesis is essential for proper intrauterine blood supply and normal growth of the fetus. A 2016 study showed that elevated testosterone levels during pregnancy can lead to disruption of the uteroplacental arterial vasculature, resulting in placental hypoxia in pregnant rats [132]. These results are consistent with findings from 2019, where hyperandrogenemia-induced poor angiogenesis was associated with a decreased embryo implantation ratio in mice [133].

It is estimated that 50–70% of PCOS women are overweight or obese [134]. According to a multicenter study conducted on 9587 ovum recipients (eliminating the oocyte as a potential confounding variable), female obesity impairs reproductive outcomes by affecting endometrial function [135]. According to a diet-based mouse model study, fatty acid accumulation in the endometrium leads to impaired decidualization and early pregnancy loss [136]. A pro-inflammatory environment, characteristic of obesity and PCOS, may cause a disruption of insulin signaling, abnormal endometrial function, and possible reproductive failures [137].

Additionally, an overall increase in androgen receptor expression in the epithelial cells of the endometrium in PCOS women was first reported in 2002 [138]. This finding has been confirmed in multiple case–control studies [139,140]. Although the levels of androgen receptors in PCOS women vary depending on the menstrual cycle [141], this phenomenon might be associated with higher miscarriage rates when combined with elevated androgen levels [142].

### 6.2. Cervix

Cervical insufficiency (CI) is higher in pregnant PCOS patients than in controls, especially among South Asian and Black women [143,144]. Furthermore, the co-occurrence of PCOS and CI is a negative factor in pregnancy [144]—a correlation was observed between high maternal androgen levels and cervical shortening during both the second and third trimesters of pregnancy, indicating cervical ripening [145]. Moreover, PCOS-affected women who received gonadotropin therapy have a higher risk of developing CI [143]. These results highlight the need for careful mid-trimester surveillance among those patients. Interestingly, the administration of Metformin did not have a statistically significant impact on cervical length in pregnant women with PCOS [145].

### 6.3. Uterine Blood Flow in Pregnant Patients with PCOS

Studies comparing blood flow in the uterine artery between pregnant women with PCOS and healthy pregnant controls are still scarce (Table 2). It is hypothesized that unfavorable hemodynamic conditions might explain frequent early pregnancy complications in women with PCOS [146].

A prospective case–control multicenter study revealed a significantly higher number of patients with PCOS were found to have abnormal uterine artery Doppler indices [147]. Furthermore, the findings suggested that the pulsatility index (PI) at the first and mid-second trimesters could be used as an independent predictor of adverse pregnancy outcomes [147]. These findings were reproduced in a 2011 study confirming significantly higher PI values in the first and late second trimesters [148]. Contrary to these results, an observational study of 172 patients, performed as a part of a randomized clinical trial, found no differences in the uterine artery pulsatility index between pregnant patients with PCOS and the healthy controls [146]. The statistical differences between groups were visible independently of the allocation to the study drug. However, the study’s methodology was poorly reported and did not use standardized measurements [146,149]

Metformin is a drug frequently used to reduce pregnancy complications in women with PCOS, even though its effectiveness remains uncertain [150]. Early small pilot studies designed to determine the effect of metformin on the uteroplacental circulation of pregnant women with PCOS, showed reduced mean uterine artery PI in women treated with metformin compared to those given a placebo [151,152]. However, a randomized, double-blind, multi-center study of 231 pregnancies showed that metformin treatment during the first trimester did not affect uterine artery blood flow evaluated using the PI [153]. Similar findings were reported in a 2018 study: metformin did not influence the PI within two hours after intake [146]. The effects of PCOS on the PI are not yet fully identified; the use of metformin within this indication remains questionable.

## 7. Discussion

This narrative review summarizes the knowledge of uterine functions and morphology in PCOS, including human and animal studies. The scope of available evidence in these topics is insufficient and must be expanded. The authors believe that both pre- and clinical trials might prove equally rewarding.

**Figure 1:**
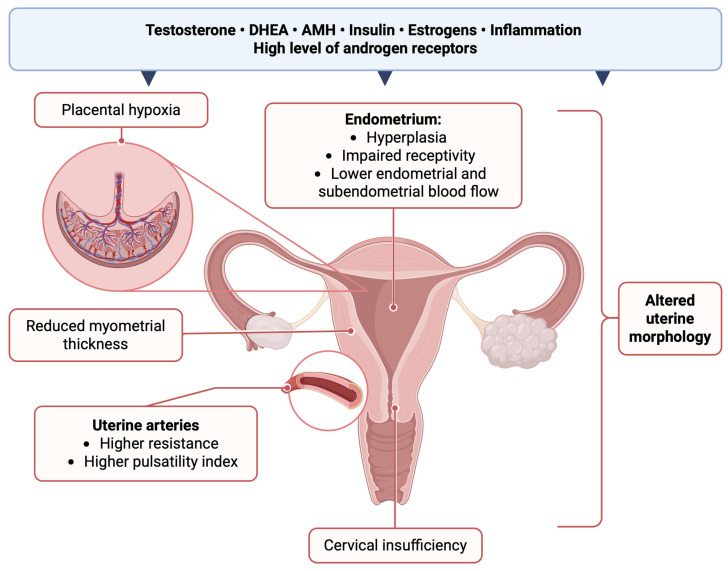
Uterine changes potentially associated with PCOS. Possible influencing factors include elevated levels of testosterone, DHEA, AMH, insulin, estrogens, associated inflammation, and high levels of androgen receptors. DHEA—Dehydroepiandrosterone; AMH—Anti-Müllerian Hormone. Created with BioRender (2025).

Currently available human studies are typically of poor quality and mostly performed retrospectively with relatively small samples and unsatisfactory reporting details. The clinical significance of available findings remains inconclusive. The non-random sampling of study groups and the heterogeneity of imaging modalities, patient characteristics, and used methodologies further restrict the generalizability of the findings. A possible link between PCOS and uterine changes reveals the need for increased surveillance in this group of patients. Altered uterine morphology, especially the higher prevalence of internal indentation, septate uterus [77], and reduced myometrial thickness [70], may potentially impact implantation rates and lead to a higher rate of miscarriages and placental dysfunction. A combination of changes in uterine morphology and suboptimal blood flow patterns within the endometrium and myometrium in the PCOS uterus may further explain a higher incidence of infertility and recurrent adverse pregnancy outcomes among PCOS patients [98,103,105,107,134,147]. The impact of PCOS on the shape of the uterus needs translation to the indirect and direct effects on tissue functions—this association requires further investigation (Figure 1).

In terms of reporting PCOS-related uterine changes, there is a need for more prospective studies with more representative patient samples for unselected women—the current scope encompasses primarily women of reproductive age treated for infertility and enrolled in specialized centers, which restricts the possible generalizability of results. Ideally, the study groups should reflect the diversity and severity of PCOS and hyperandrogenism in the general population. Thus, the sampling method should preferably select random or non-random consecutive individuals to assess the occurrence of PCOS and/or hyperandrogenism (study group) or lack thereof (control group). The conducted research should also reflect morphology and functions in different age groups. It is vital for both the control and investigated groups to undergo the same endocrinological assessment to exclude any confounding variables or undiagnosed hormonal disorders [16]. Future studies should explore the impact of prolonged hormonal disturbance on the function and shape of uterine muscle and cavity, preferably by performing detailed measurements using 2D and 3D ultrasound [77,79].

The available animal studies explore mechanisms of uterine changes observed in PCOS, demonstrating the pathophysiology of excess androgen stimulation in utero and prolonged exposure following menarche. Rodent models point to an increased uterine mass and dimensions, with decreasing quality of the endometrial and myometrial tissue, resulting in implantation disruptions and genetic dysregulations [109,110,111,112]. Model studies allow for a more exhaustive analysis of the complex influence of metabolic changes associated with PCOS and their influence on the development of uterine and placental defects [114,115,116]. However, the animal models may often offer results contradictory to those of human trials, and as such, should be extrapolated with caution. Future studies should focus on the differentiation between prenatal and postnatal influences of PCOS on the reproductive tract.

## 8. Conclusions

Multiple studies have described PCOS-related abnormalities, but only a few have reliably assessed uterine alterations and their clinical significance. Outlining the potential impact of hormonal imbalance on the structure and function of the uterus is essential to establishing proper management in affected women, including the improvement of reproductive outcomes. Further research is needed to explore possible associations between the levels of androgens, uterine morphology, and the prevalence of acquired or congenital uterine changes. Future studies should include large prospective cohorts utilizing detailed 3D ultrasound measurements and extensive hormone profiling.

## Figures and Tables

**Table 1:** Uterine blood flow in non-pregnant patients with PCOS.

Parameter	Conclusions	Study Type ^a^	Population	Values in PCOS Patients vs. in Non-PCOS Patients	*p* Value	Author
Endometrial and subendometrial blood flow	Higher in PCOS patients	Prospective case–control	67 patients (37 PCOS vs. 30 healthy controls)	Endometrial pulsatility index (PI)—mean ± SD Follicular phase: 0.96 ± 0.13 vs. 0.72 ± 0.18 Peri-ovulatory phase: 0.97 ± 0.17 vs. 0.83 ± 0.13 Luteal phase: 1.00 ± 0.15 vs. 0.80 ± 0.13 Endometrial resistance index (RI)—mean ± SD Follicular phase: 0.57 ± 0.11 vs. 0.48 ± 0.10 Peri-ovulatory phase: 0.62 ± 0.12 vs. 0.55 ± 0.11 Luteal phase: 0.636 ± 0.10 vs. 0.54 ± 0.12 Subendometrial pulsatility index (PI)—mean ± SD Follicular phase: 0.90 ± 0.13 vs. 0.65 ± 0.16 Peri-ovulatory phase: 0.91 ± 0.11 vs. 0.76 ± 0.12 Luteal phase: 0.90 ± 0.14 vs. 0.77 ± 0.12 Subendometrial resistance index (RI)—mean ± SD Follicular phase: 0.54 ± 0.10 vs. 0.47 ± 0.10 Peri-ovulatory phase: 0.56 ± 0.10 vs. 0.54 ± 0.11 Luteal phase: 0.58 ± 0.08 vs. 0.53 ± 0.12	*p* < 0.05 *p* < 0.05 *p* < 0.05 *p* < 0.05 *p* < 0.05 *p* < 0.05 *p* < 0.05 *p* < 0.05 *p* < 0.05 *p* < 0.05 *p* < 0.05 *p* < 0.05	Palomba et al., 2006 [94]
	No difference between PCOS and control groups ^b^	Prospective observational	72 patients (36 PCOS vs. 36 controls from the assisted conception unit and the infertility clinic)	Endometrial parameters—median (range) Follicular phase: VI% 0.60 (0.06–1.45) vs. 0.86 (0.23–1.76) FI 31.57 (23.54–50.76) vs. 32.14 (24.21–69.41) VFI 0.21 (0.02–0.56) vs. 0.28 (0.07–0.65) Subendometrial parameters—median (range) Follicular phase: VI% 1.92 (0.16–5.17) vs. 2.47 (0.85–5.29) FI 39.98 (30.72–56.17) vs. 39.09 (34.66–67.08) VFI 0.76 (0.07–2.01) vs. 0.96 (0.35–2.48)	*p* ≥ 0.05 *p* ≥ 0.05 *p* ≥ 0.05 *p* ≥ 0.05 *p* ≥ 0.05 *p* ≥ 0.05	Lam et al., 2009 [93]
Uterine artery pulsatility index (PI)	Higher in PCOS patients	Prospective	103 patients (88 PCOS vs. 15 patients with normal menstrual cycle)	Mean ± SD 2.97 ± 0.9 vs. 1.89 ± 0.2	*p* < 0.05	Ajossa et al., 2001 [100]
		Prospective case–control	67 patients (37 PCOS vs. 30 healthy controls)	Mean ± SD Follicular phase: 3.41 ± 0.97 vs. 2.37 ± 0.54 Peri-ovulatory phase: 3.30 ± 1.02 vs. 2.76 ± 0.41 Luteal phase: 3.32 ± 0.96 vs. 2.81 ± 0.54	*p* < 0.05 *p* < 0.05 *p* < 0.05	Palomba et al., 2006 [94]
		Prospective controlled	Not reported		*p* < 0.05	Vrtačnik-Bokal et al., 2006 [102]
		Prospective	97 patients (55 PCOS vs. 42 healthy controls)	Mean ± SD 4.88 ± 0.96 vs. 4.11 ± 0.82	*p* < 0.01	Adali et al., 2009 [103]
		Prospective case–control	50 patients (25 PCOS vs. 25 healthy controls)	Mean ± SD 3.74 ± 1.01 vs. 2.43 ± 0.36	*p* < 0.001	Mala et al., 2009 [101]
	No difference between PCOS and control groups	Prospective case–control	22 patients (10 PCOS vs. 12 controls)	Mean ± SD 2.9 ± 1.2 vs. 3.1 ± 0.8	*p* > 0.05	Pinkas et al., 1998 [98]
		Prospective observational	72 patients (36 PCOS vs. 36 controls from the assisted conception unit and the infertility clinic)	Median (range) 2.48 (1.29–4.60) vs. 2.52 (1.24–7.04)	*p* ≥ 0.05	Lam et al., 2009 [93]
Uterine artery resistance index (RI)	Higher in PCOS patients	Prospective case–control	67 patients (37 PCOS vs. 30 healthy controls)	Mean ± SD Follicular phase: 0.96 ± 0.12 vs. 0.67 ± 0.10 Peri-ovulatory phase: 0.96 ± 0.14 vs. 0.84 ± 0.10 Luteal phase: 0.98 ± 0.14 vs. 0.84 ± 0.12	*p* < 0.05	Palomba et al., 2006 [94]
		Prospective controlled	49 patients (18 PCOS and 31 women with normal menstrual cycle)	Mean ± S.E. 0.90 ± 0.05 vs. 0.86 ± 0.06	*p* < 0.05	Vrtačnik-Bokal et al., 2006 [102]
		Prospective case–control	50 patients (25 PCOS vs. 25 healthy controls)	Mean ± SD 0.87 ± 0.04 vs. 0.80 ± 0.06	*p* < 0.001	Mala et al., 2009 [101]
	No difference between PCOS and control group	Prospective case–control	22 patients (10 PCOS vs. 12 controls)	Mean ± SD 0.92 ± 0.10 vs. 0.91 ± 0.07	*p* > 0.05	Pinkas et al., 1998 [98]
		Prospective observational	72 patients (36 PCOS vs. 36 controls from the assisted conception unit and the infertility clinic)	Median (range) 0.86 (0.68–0.99) vs. 0.87 (0.70–0.99)	*p* ≥ 0.05	Lam et al., 2009 [93]
		Case–control	77 patients (45 PCOS vs. 32 healthy controls)	Mean ± SD 0.96 ± 0.42 vs. 0.87 ± 0.13	*p* ≥ 0.05	Younesi et al., 2019 [99]

^a^ As declared by the original study authors. ^b^ The study authors reported significantly reduced endometrial and subendometrial blood flow in anovulatory and hyperandrogenic patients with PCOS, as compared to the control group. Additionally, ovulatory women with hyperandrogenism had significantly lower blood flow than their normoandrogenic but anovulatory controls. The authors analyzed average RI of the uterine arteries on both sides, as the findings of the two sides showed no significant difference in the statistical scale. VI—vascularization index, FI—flow index, VFI—vascularization flow index.

**Table 2:** Uterine blood flow in pregnant patients with PCOS.

Parameter	Conclusions	Study Type ^a^	Population	Values in PCOS Patients vs. in Non-PCOS Patients	*p* Value	Author
Uterine artery pulsatility index (PI)	Higher rate of abnormal PI	Prospective case–control	139 patients (70 PCOS vs. 69 healthy controls)	n (%) Baseline: 42 (60.0) vs. 29 (42.0) 8 hbd: 39 (55.7) vs. 27 (39.1) 10 hbd: 38 (34.3) vs. 26 (37.7) 12 hbd: 36 (51.4) vs. 24 (34.8) 20 hbd: 36 (51.4) vs. 23 (33.3)	*p* < 0.05 *p* < 0.05 *p* < 0.05 *p* < 0.05 *p* < 0.05	Palomba et al., 2010 [142]
	Higher PI in PCOS patients	Prospective case–control	80 patients (40 ovulatory PCOS vs. 40 healthy controls) ^b^	Mean ± SD 8 hbd: 2.93 ± 1.02 vs. 2.4 ± 0.95 12 hbd: 2.71 ± 1.03 vs. 1.9 ± 0.89 26 hbd: 1.9 ± 1.01 vs. 1.4 ± 0.93	*p* = 0.014 *p* = 0.001 *p* = 0.024	Nouh and Shalaby, 2011 [143]
	No difference between PCOS/healthy patients	Prospective observational	142 patients (24 PCOS vs. 118 healthy controls) ^c^	Mean (CI) 1.80 (1.58–2.03) vs. 1.79 (1.70–1.87)	*p* = 0.18	Stridsklev et al., 2018 [141]

^a^ As declared by the original study authors. ^b^ At 12 hbd and 26 hbd, the number of PCOS patients was 32 and 39, respectively. ^c^ Data presented for PCOS placebo patients at inclusion of the study. Both groups were non-fasting. CI—confidence interval, hbd—gestational week.

## Data Availability

The data underlying this article is available in the article.

## Abbreviations

The following abbreviations are used in this manuscript:

PCOS	Polycystic ovary syndrome
2D/3D	Two- or three-dimensional
CI	Cervical insufficiency
TVUS	Transvaginal ultrasound
DHT	5α-dihydrotestosterone
INS	insulin
ROS	Reactive oxygen species
BMI	Body mass index
DHEAS	Dehydroepiandrosterone sulfate
PI	Artery pulsatility index
RI	Resistance index
LH	Luteinizing hormone
FSH	Follicle-stimulating hormone
AMH	Anti-Müllerian Hormone
DES	Diethylstilbestrol
ASRM	American Society for Reproductive Medicine
ESHRE	European Society of Human Reproduction and Embryology
SHBG	Sex hormone-binding proteins
